# Prevalence of *Helicobacter pylori* and its CagA subtypes in gastric cancer and duodenal ulcer at an Austrian tertiary referral center over 25 years

**DOI:** 10.1371/journal.pone.0197695

**Published:** 2018-05-29

**Authors:** Yumiko Kamogawa-Schifter, Yoshio Yamaoka, Tomohisa Uchida, Andrea Beer, Barbara Tribl, Maximilian Schöniger-Hekele, Michael Trauner, Werner Dolak

**Affiliations:** 1 Division of Gastroenterology and Hepatology, Department of Internal Medicine III, Medical University of Vienna, Vienna, Austria; 2 Department of Environmental and Preventive Medicine, Oita University Faculty of Medicine, Yufu-city, Oita, Japan; 3 Department of Molecular Pathology, Oita University Faculty of Medicine, Yufu-city, Oita, Japan; 4 Department of Pathology, Medical University of Vienna, Vienna, Austria; National Cancer Center, JAPAN

## Abstract

**Background and aims:**

The prevalence of *Helicobacter pylori (H*. *pylori)* tends to be lower in Western countries such as central Europe compared with Asia. The virulence of *H*. *pylori* is influenced by its subtype composition, most importantly by the presence or absence of different types of cytotoxin-associated gene A(CagA). This study aimed to assess the prevalence of *H*. *pylori* and its respective CagA phenotype in a large retrospective cohort of patients with gastric cancer or duodenal ulcer at a Western tertiary referral institution.

**Methods:**

*H*. *pylori* positive gastric biopsy samples from patients diagnosed with the afore mentioned diseases within the past 25 years were re-evaluated by histology for *H*. *pylori* and status of gastritis. Confirmed *H*. *pylori* positive cases were processed for immunohistochemistry (IHC) for *H*. *pylori*,CagA, and EastAsiantype CagA.

**Results:**

The prevalence of *H*. *pylori* positive gastric biopsy samples decreased from 20.7% to 2.3% within the study period. Among the gastric cancer patients, the *H*. *pylori* positive rate was 16.6%, and didn’t show significant changes over time (p = 0.38). Contrary, the *H*. *pylori* positive rate of duodenal ulcer decreased significantlyfrom 40% to 5% (p = 0.01). Within *H*. *pylori* positive groups ofboth diseases, CagA was highly detected at IHC (86% and 78%, respectively). Except for a few patients originating from East Asian countries, all CagA detected in this study were of Western type.

**Conclusion:**

In this first Western investigation on the chronological prevalence of *H*. *pylori* and its most relevant subtypes, Western type of CagA was highly detected in two important index diseases of the pathogen. This raises further questions about the virulence of this subtype.

## Introduction

*Helicobacter pylori (H*. *pylori*) is a Gram-negative spiral-shaped bacterium that colonizes the stomach and triggers various gastric diseases such as peptic ulcer, chronic gastritis, and eventually gastric malignancy in human [1]. Epidemiological studies revealed that more than 50% of the human population are infected with *H*. *pylori*. However, infection prevalence shows large geographical variations [2]. In general, the prevalence of infection is higher in developing countries than developed countries such as Europe and North America [3]. In Austria, for example, the prevalence rate of *H*. *pylori* infection was found to be 23% in a recent study [4].

In 1994, the International Agency for Research on Cancer (IARC) of the World Health Organization declared *H*. *pylori* to be a group I human carcinogen for gastric adenocarcinoma [5]. Although infection is common among humans, only a subset of infected population develops peptic ulcer diseases (10–20%) and distal gastric adenocarcinoma(1–2%) [6]. Since not all infected individuals develop gastric cancer, other factors such as host-related (genetic), environmental, and bacterial virulence factors, such as CagA, VacA, OipA, are considered to be responsible for a neoplastic process.

The cytotoxin-associated gene A (CagA) protein is the most extensively studied virulent factor of *H*. *pylori*. CagA is encoded by the *cagA*gene. The*cagA*is located in the end of the *cag*pathogenecity island (PAI), which functions as type IV secretion system and is thought to be introduced into the *H*. *pylori* genome from an unknown organism [7]. Two types of *H*. *pylori* (CagA-producing, CagA-non-producing) strains were isolated. Several animal studies showed that only CagA positive strains, but not CagA negative strains of *H*. *pylor*i infection developed gastric malignancy [8,9].

The worldwide infection ratio of CagA positive and CagA negative strains is about 6:4, except for the East Asia region, where most strains are CagA positive. It has been reported that individuals infected with CagA positive strains of *H*. *pylori* are at a higher risk of peptic ulcer or gastric cancer than those infected with CagA negative strains (5.8-fold vs. 2.5-fold and 3.4-fold vs. 1.2-fold, respectively)[10–12].

*cagA*is a polymorphic gene, especially in its C-terminal region [13]. It was found that most of the Western CagA consist of Glu-Pro-Ile-Tyr-Ala (EPIYA)- A, B, C, whereas Eastern CagA contain EPIYA-A, B and D instead of C. Almost all East Asian strains contain a single EPIYA-D segment and have a greater ability to induce morphological changes in epithelial cells than Western types [13,14]. Thus, virulence of *H*. *pylori* may at least some part depend on the CagA phenotype.

The aim of this study was to assess the prevalence of *H*. *pylori* and its respective CagA phenotype in a large retrospective cohort of patients with gastric cancer or duodenal ulcer as a non-cancer control at a Western tertiary referral institution.

## Materials and methods

This study was conducted at the Medical University of Vienna, Austria in close cooperation with the Oita University Faculty of Medicine, Japan. The study protocol was approved by the internal review board (IRB) of the Medical University of Vienna (EK 1706/2015). The IRB waived the need for consenting patients about the analysis of their historical samples. Prior to analysis, all patient data had been de-identified using pseudonymization.

### Identification of study samples

Patients who were diagnosed with gastric cancer or duodenal ulcer by endoscopy performed at the Division of Gastroenterology and Hepatology, Department of Internal Medicine III, Medical University of Vienna (the largest tertiary referral center in Austria) between 1991 and 2015 were identified by reviewing the electronic database of endoscopic examination reports. Patients’ data were analyzed to assess demographics, location of the cancer and status of *H*. *pylori* infection by histology (*H*. *pylori* infection is routinely examined at the study institution in all patients undergoing upper gastrointestinal endoscopy by using modified Giemsa staining of four gastric biopsy samples–from the antrum and body of the stomach).

### *H*. *pylori* and CagA analysis

In the context of this study, all *H*. *pylori* positive cancer samples of patients that had been identified as outlined above were re-evaluated histologically at the study institution. The same sample size was randomly selected (according to a computer-generated list) from the population of*H*. *pylori* positive duodenal ulcer samples which served as non-cancer controls.This selection also underwent histological re-evaluation. In case of histological confirmation of *H*. *pylori* infection, samples from both groups (gastric cancer and duodenal ulcer) were shipped to the cooperating institution in Japan to perform immunohistochemistry (IHC) for *H*. *pylori*,CagA, and East Asian type of CagA as previously described [15]. In brief, after antigen retrieval and inactivation of endogenous peroxidase activity, samples were incubated with anti-*H*. *pylori* antibody (DAKO, Denmark), anti-CagA antibody (b-300 Santa Cruz, USA), or α-EAS antibody [15]at 4°C forovernight, and followed by biotinylated goat anti-rabbit or anti-rat IgG (Nichirei Co., Japan). The bacterial load was classified into four grades: 0, none; 1, mild; 2, moderate; and 3, marked according to the updated Sydney system [16]. Then bacterial loads equal to or greater than 1 with positive results from IHC were considered *H*. *pylori* positive.

### States of gastritis

Status of gastritis was also re-evaluated by histology. It was determined by the degree of neutrophil infiltration(activity), lymph-mononuclear cell infiltration(inflammation) atrophy, intestinal metaplasia according to the updated Sydney system: 0, ‘normal’; 1, ‘mild’; 2, ‘moderate’; and 3, ‘marked’ [16]. Chronic gastritis was defined by aninflammation score equal to or greater than 1. Active gastritis was determined as a neutrophil score equal to or greater than 1. Gastritis stage was assessed based on topographic locations (antrum and corpus), according to the Operative Link on Gastritis Assessment (OLGA) system [17].

### Statistical analysis

Number of gastroscopies per year and rate of *H*.*pylori*infection over the study period were reported descriptively. Number of patients diagnosed with gastric cancer or duodenal ulcer were extracted. The rate of *H*.*pylori* infection was reported for both diseases and evaluated for significant changes over time using linear regression analysis. Location of gastric cancer was compared between *H*. *pylori* positive and negative subset using Fisher’s exact test. Histological features of gastritis such as intestinal metaplasia and atrophy were compared between both diseases for the two different gastric biopsy locations.

A subset of study samples in which *H*.*pylori*positivity could beconfirmed by histological re-evaluation was processed for IHC as outlined above. Results of IHC for *H*. *pylori*, CagA and East Asian type of CagA were reported and compared between both diseases using Fisher’s exact test.A p-value <0.05was considered statistically significant. All data analyses were performed using SPSS (version 23.0). All co-authors had access to the study data and reviewed and approved the final manuscript.

## Results

**Fig 1:**
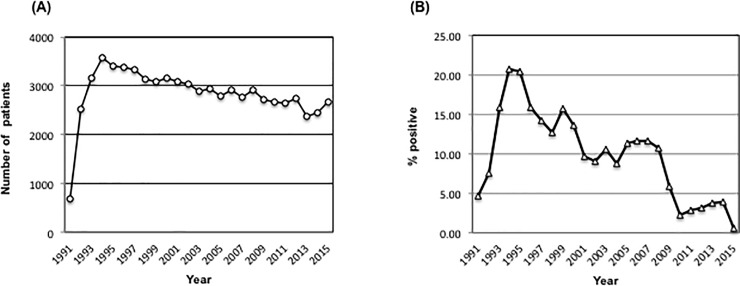
The prevalence of *H*. *pylori* infection. (A) The number of patients who underwent upper gastrointestinal endoscopy per year. (B) Percentage of *H*. *pylori* positive patients per year.

From 1991 to 2015, a total of 70997 upper GI endoscopies (average 2840/year) had been performed at the study institution (Fig 1A). As shown in Fig 1B, the *H*. *pylori* positive rate examined by histological examination was 4.7%at the beginning and, then gradually increased up to 20.7% in 1994. In 2000, this rate again decreased to 10%, and stayed in that range for the next eightyears. After 2010, the positive rate of *H*. *pylori* markedly decreased to 2.3%.

**Fig 2:**
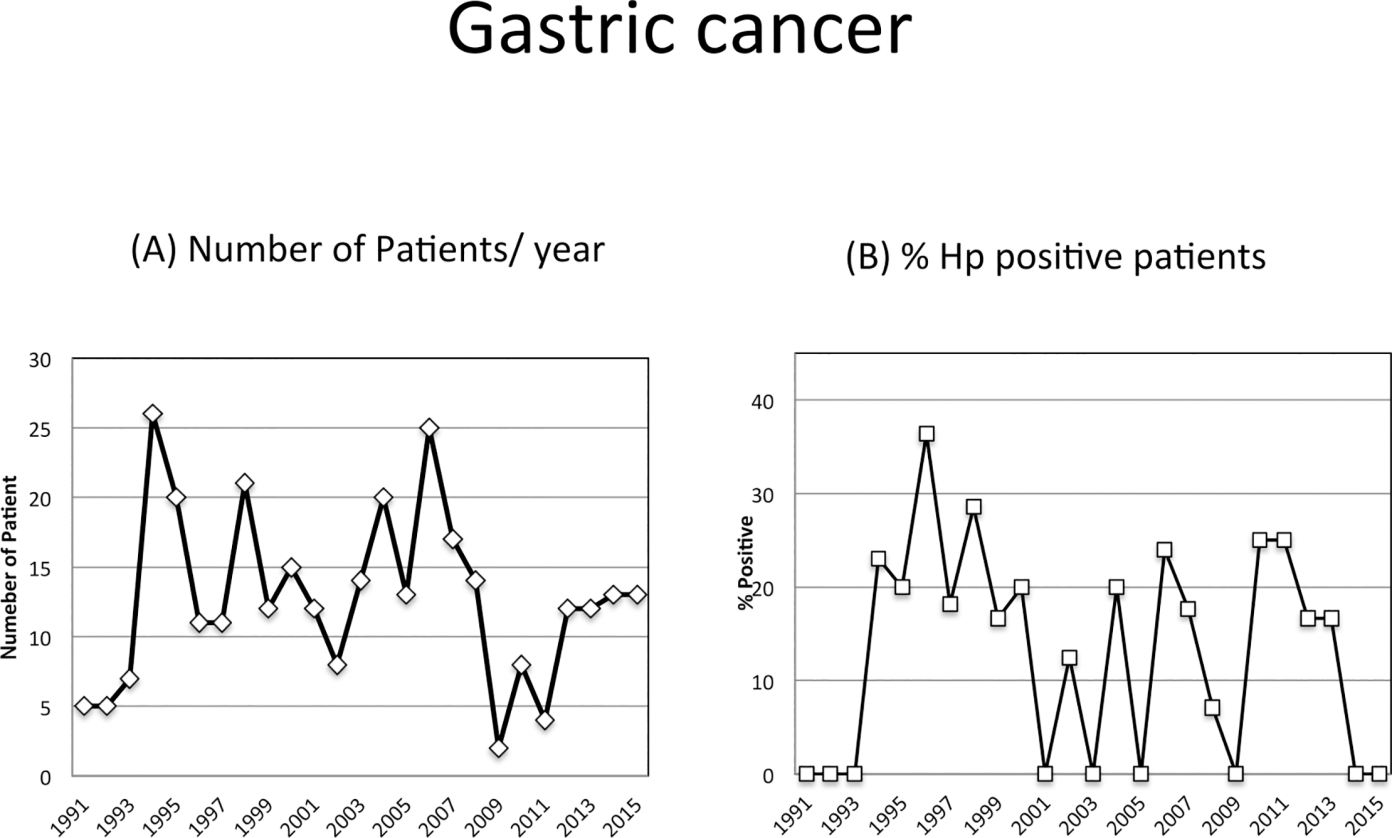
The incidence of gastric cancer. (A) Total number of gastric cancer patients per year.(B) Percentage of *H*. *pylori* positive patients with gastric cancer per year.

**Fig 3:**
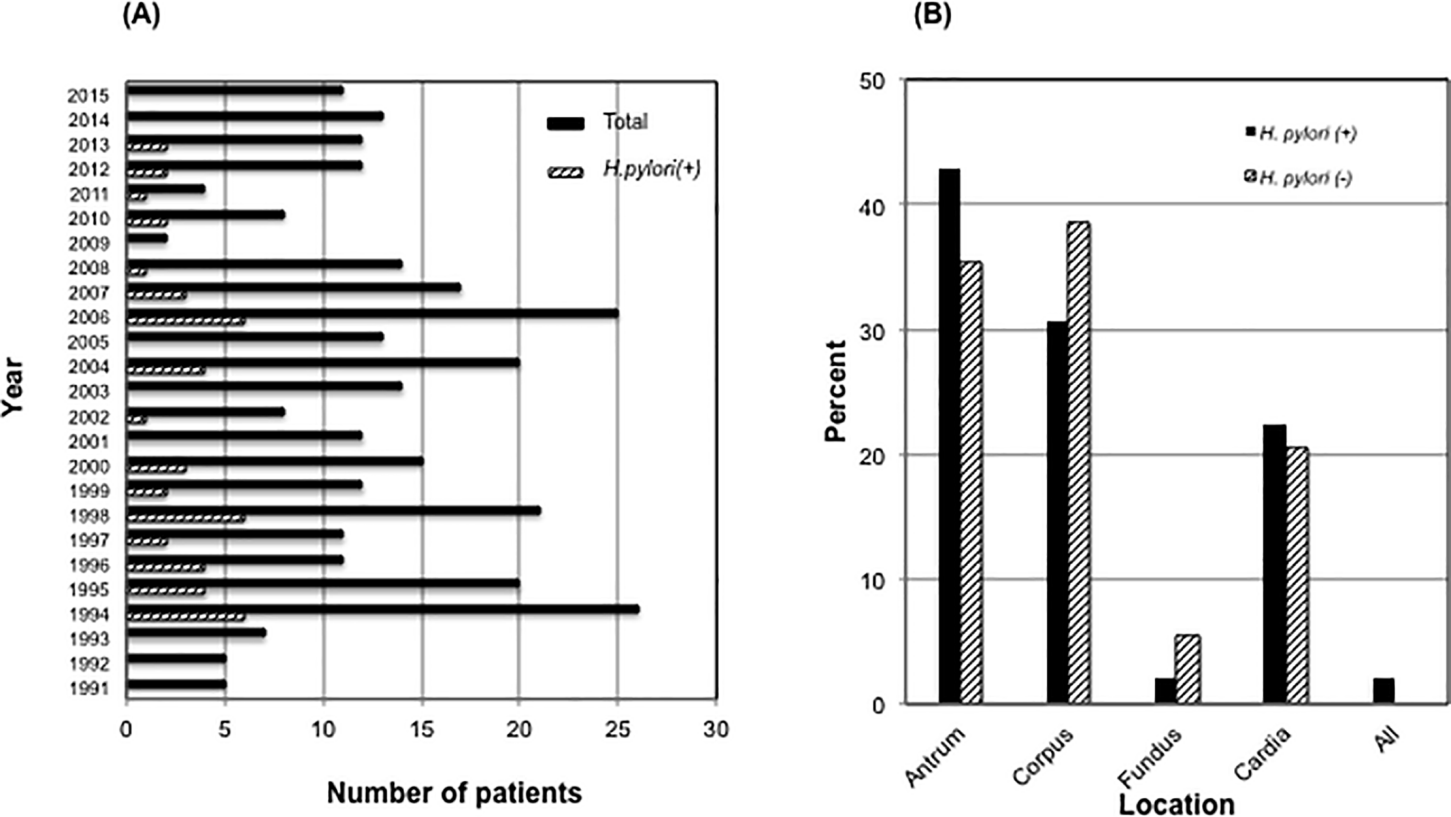
The incidence and location of gastric cancer. (A) Black bar indicates number of gastric cancer patients per year. Shaded bar indicates *H*. *pylori* positive gastric cancer patients per year.(B) The location of the cancer in patients either *H*. *pylori* infected(black bar) or non-infected(shaded bar).

Within 25 years, the total number of primary gastric cancer patients was 309 (median age 69 years,range 28–95 years; 183 males). The amount of cases diagnosed per yeardid not show a significant change over time (p = 0.19; Fig 2A).90% of cancerswere found in advanced stage. According to histology, the positive rate of *H*. *pylori* in gastric cancer was 16.6% and did neithershow significantchanges during the observed period (p = 0.38; Figs 2B and 3A).

As shown in Fig 3B the localization of cancer was highest in the corpus (37%) and the antrum (36%). The total amount of non-cardiac cancer was 78.6%. There was a significant difference of cancer location between *H*. *pylori* positive and *H*. *pylori* negative patients (p = 0.03): In *H*. *pylori* positive cases the tumor was mostly located in the antrum followed by the body of the stomach, whereas it was the other way round in *H*. *pylori* negative cases.

**Fig 4:**
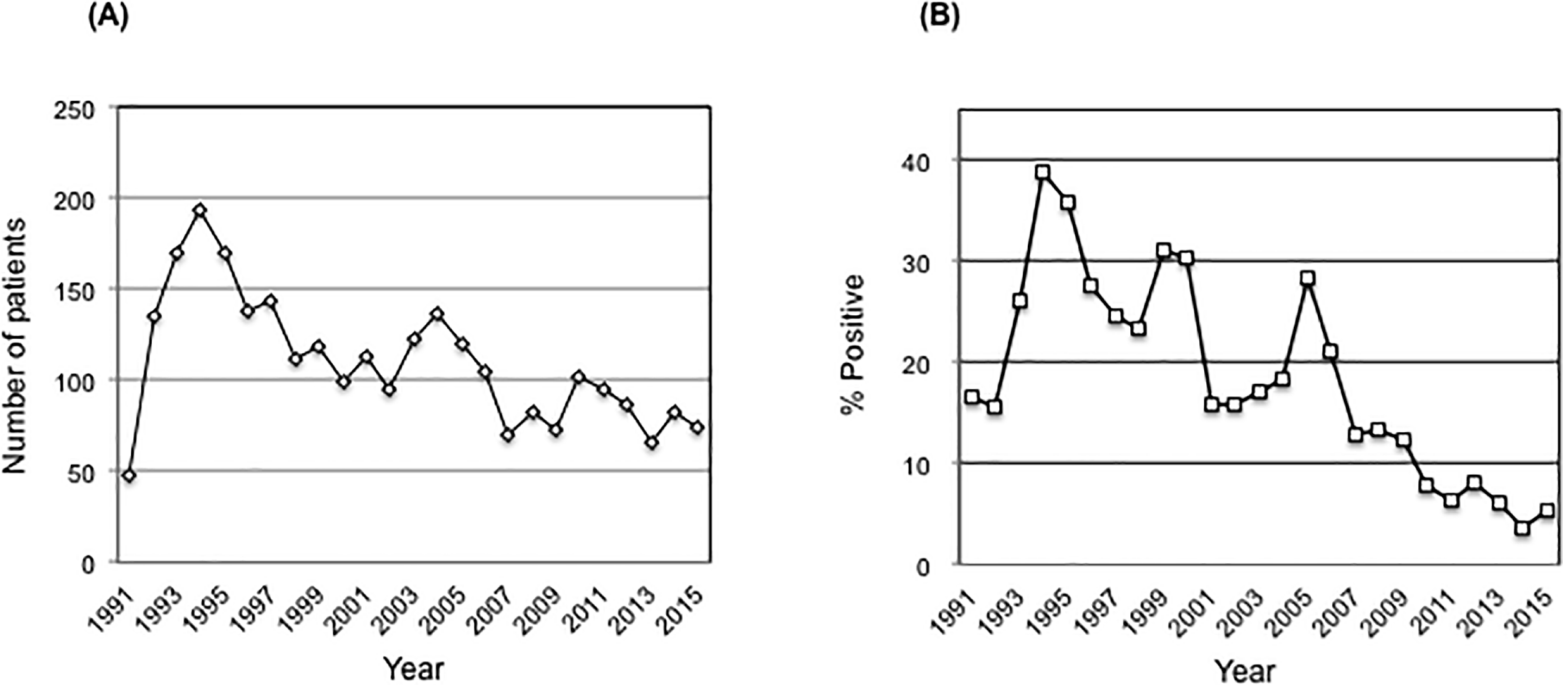
The incidence of duodenal ulcer. (A) Total number of duodenal ulcer patients per year.(B) Percentage of *H*. *pylori* positive patients with duodenal ulcer per year.

Within the same time frame, the total number of patients diagnosed with duodenal ulcer was 2747 (median age 56 years, range 18–87 years, 1798 males). Among all duodenal ulcer patients, 20.8% of them were *H*. *pylori* positive. Both, the total number of duodenal ulcer patients and the rate of H. pylori positive cases among them decreased significantly during the observed period (p = 0.03 and p = 0.01, respectively; Fig 4).

**Fig 5:**
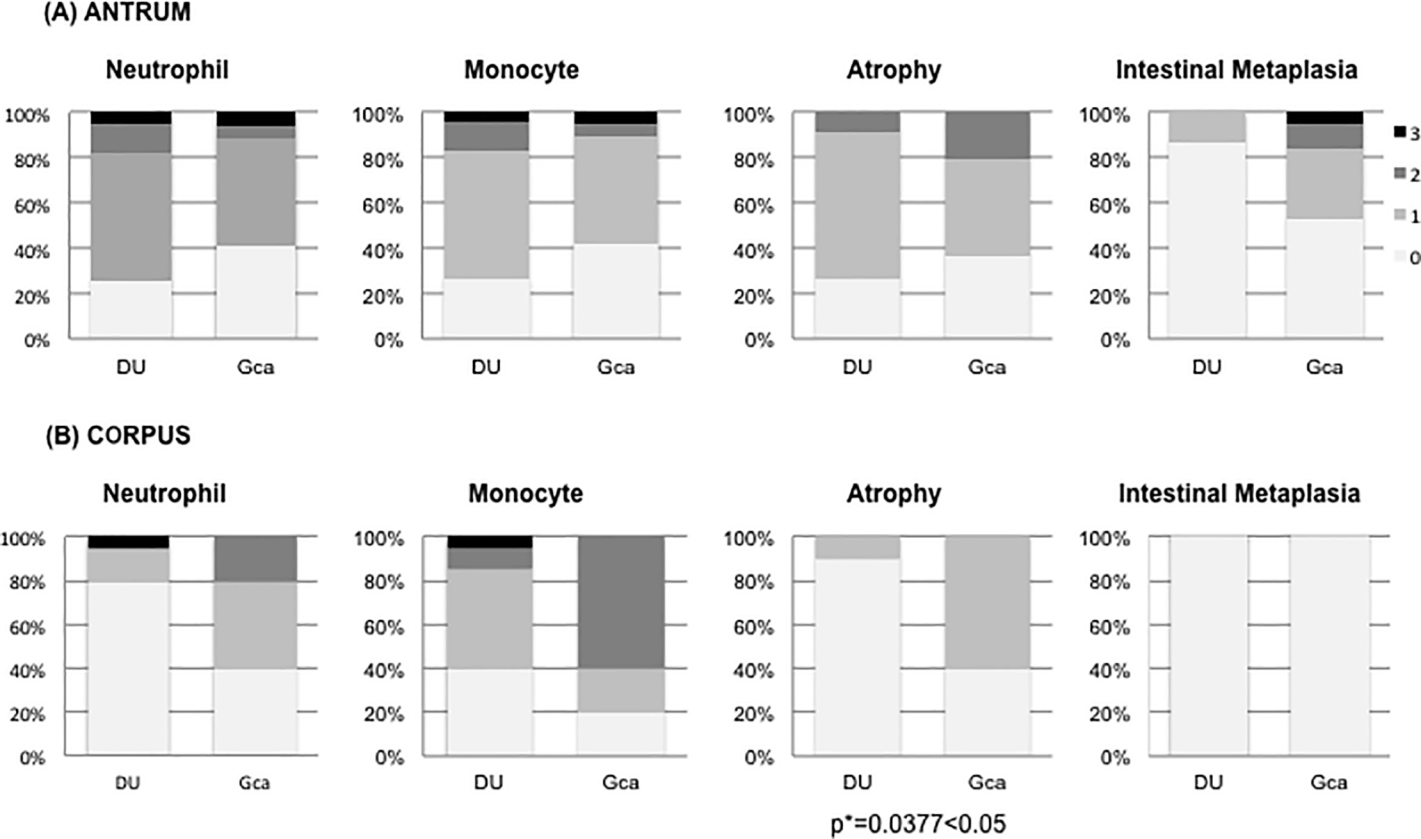
Status of gastritis by histology. Activity, inflammation, atrophy and intestinal metaplasia in both the antrum and the corpus in duodenal ulcer patients and gastric cancer patients. (0, ‘normal’, white bar; 1, ‘mild’,grey bar; 2, ‘moderate’, dark grey bar;and 3, ‘marked’, black bar).

The histological features of gastritisaccording to gastric location are shown in Fig 5 for both gastric cancer and duodenal ulcer patients. For both diseases, gastritis showed similar activities, inflammation, and atrophy in the antrum. In the antrum, the presenceof intestinal metaplasia seemed to be higher in gastric cancer patients than in duodenal ulcer patients.However, the difference was not significant (p = 0.09). In the corpus, atrophy was significantly more frequent in gastric cancer patients than in duodenal ulcer patients (p = 0.04).

In total, 63 *H*. *pylori* positive samples (24 cases of duodenal ulcer and 39 cases of gastric cancer) were confirmed by histological re-evaluation asoutlined above and processed for IHC. However, IHC could not detect *H*. *pylori* in all the samples, most probably due to the condition of the old samples. As shown in Table 1, most *H*. *pylori*deriving from patients with duodenal ulcer (78%) and gastric cancer (86%) expressed CagA. The types of CagA were mostly non-East Asian type in both diseases. Only a few patients showed East Asian type CagA (gastric cancer 7%, duodenal ulcer 17%). There was no significant difference in the frequency of both, CagA and East Asian type CagA between the two diseases studied (p = 0.89 and p = 0.37, respectively).

**Table 1:** CagA status in samples proven to be *H*. *pylori* positive by immunohistochemistry.

	H. pylori(+)	CagA (+)	East AsianCagA(+)
**Gastric cancer**	14	86%	7%
**Duodenal ulcer**	18	78%	17%

More detailed characteristics of *H*. *pylori* positive patients at IHC are displayed in Tables 2 and 3 as well as in S1 Table. Male gender was predominant in this study. All patients with East Asian type CagA originated from Asian countries.

**Table 2:** Characteristics of *H*. *pylori* positive gastric cancer patients confirmed by immunohistochemistry.

		CagA -		CagA +			
				Non-EA Cag A +		EA CagA +	
		N	%	N	%	N	%
Number of patients		2	14	11	79	1	7
Median Age (range) in years		46 (36–55)		59(47–83)		41	
Sex	male	2	14	8	57	1	7
	female	0	0	3	21	0	0
Country of	Austria	2	14	6	43	0	0
origin	Yugoslavia	0	0	3	21	0	0
	Turkey	0	0	2	14	0	0
	China	0	0	0	0	1	7
Indication for	Abdominal pain	0	0	3	21	0	0
gastroscopy	Bleeding	1	7	3	21	0	0
	Anemia	0	0	1	7	0	0
	Loss of appetite	0	0	0	0	1	7
	Cancer screening	1	7	4	29	0	0
Concomitant	liver disease	0	0	0	0	0	0
Concomitant	antibiotics	1	7	2	14	0	0

CagA = cytotoxin-associated gene A; EA CagA = East Asian type cytotoxin-associated gene A

**Table 3:** Characteristics of *H*. *pylori* positive duodenal ulcer patients confirmed by immunohistochemistry.

		CagA -		CagA +			
				Non-EA Cag A +		EA CagA +	
		N	%	N	%	N	%
Number of patients		4	22	11	61	3	17
Median age (range) in years		41(20–84)		56 (24–73)		28 (19–32)	
Sex	male	3	17	7	39	3	17
	female	1	6	4	22	0	0
Country of	Austria	1	6	7	39	0	0
origin	Yugoslavia	3	17	1	6	0	0
	Kazakhstan	0	0	1	6	0	0
	Turkey	0	0	1	6	1	6
	Egypt	0	0	1	6	0	0
	China	0	0	0	0	2	11
Indication for	Abdominal pain	1	6	6	33	2	11
gastroscopy	Bleeding	1	6	1	6	1	6
	Anemia	0	0	1	6	0	0
	Loss of appetite	0	0	1	6	0	0
	Cancer Screening	2	11	2	11	0	0
Concomitant	liver disease	0	0	2	11	0	0
Concomitant	antibiotics	1	6	2	11	0	0

CagA = cytotoxin-associated gene A; EA CagA = East Asian type cytotoxin-associated gene A

## Discussion

This is the first study that investigated the prevalence of *H*. *pylori* infection at a major Austrian tertiary referral hospital serving a large population of Austria over a long period (25 years). The total prevalence of *H*. *pylori* was much lower than previously reported(23–50%) [4,18,19]. The reason is not completely clear but might be partially influenced by a selection bias since the study institution is a tertiary referral center specialized for the treatment of patients with liver diseases (who mostly have a high chance to be treated with antibiotics during the course of their disease). Whereas the total number of patients increased at the beginning of the study period due to expansion of the institution, the total number of *H*.*pylori* diagnoses was very low and showed a rapid increase within the first three years of observation. This might be caused by a learning process among pathologists which had been reported previously [20,21].

The *H*. *pylori* infection rate remarkably decreased within the observed period which might be attributed to the increasing awareness for the pathogen as well as a widespread of eradication therapy for *H*. *pylori* infection. During the same time frame the incidence of duodenal ulcer showed an almost similar course. This evidence fits with the fact that *H*. *pylori* implicates as a major etiologic factor in duodenal ulcer [22,23]. However, the average *H*. *pylori* -positive rate in duodenal ulcer in the observed sample (21%) is much lower than previously reported (80 to 95%) [24]. Since the average age of duodenal ulcer patients in that study is higher than usual, we may have seen more patients whose ulcer could have been caused by other confounding factors, such as use of NSAIDs, steroids, or anti-coagulants.

Contrary to *H*. *pylori* positive duodenal ulcer, the incidence of *H*. *pylori* positive gastric cancer remained stable within the observed period. *H*. *pylori* is recognized as a group 1 human carcinogen, and epidemiologic studies showed a six-fold increased risk of gastric cancer in *H*. *pylori* infected populations compared with uninfected populations [25]. However, it may take longer to develop gastric cancer than duodenal ulcer, therefore the *H*. *pylori* positive rate might not have changed during the study period. In addition, it is reported that *H*. *pylori* is hard to detect at the late stage of *H*. *pylori* infection.

Although it is well known that the frequency of gastric cancer depends on the oncogenic *H*. *pylori* infection [26, 27], the actual *H*. *pylori* positive rate in gastric cancer patients was very low in this study (16%). It was much lower than previously reported (3) which can be explained as follows: Many cancers might have arisen in patients who were previously infected with *H*. *pylori* for a long time. However, at the time point of cancer diagnosis, the pathogen couldn’t be detected any longer due to previous antibiotic therapy, late stage *H*. *pylori* infection or extensive tumor growth without remaining tissue of normal gastric mucosa.

Gastric cancer is believed to develop based on chronic gastritis [28]. We have re-examined samples for the status of gastritis and confirmed that both duodenal ulcer and gastric cancer infected with *H*. *pylori* revealed active and chronic gastritis. In addition, gastric cancer patients showed significantly higher atrophy in the corpus than duodenal ulcer patients. According to a report by Uemura et al [29], atrophic changes and intestinal metaplasia are strongly related to the risk of gastric cancer. This investigation also confirmed this hypothesis. Thus, investigation of atrophy in the corpus is very important for cancer screening.

Another risk factor for gastric cancer is the virulence of *H*. *pylori*. *H*. *pylori* is a highly heterogeneous bacterium. Its diversity seems to contribute to the outcome of the disease [30,31]. The existence of CagA is associated with gastric cancer [10,11,32] and peptic ulcers [12]. The results of the present study confirmed the importance of CagA for both diseases. Recent studies showed that East Asian type CagA is more virulent than Western type CagA in relation to clinical outcome [31, 33]. Interestingly, only a minority of our study patients was found to have East Asian type CagA in both diseases and all of them were Asian immigrants. This suggests that also Western type CagA is toxic to develop gastric diseases and is strictly fixed with its geography.

This study has several limitations. Besides the selection bias that was addressed already, the analysis of samples that might have been stored for up to 25 years is suboptimal. Accordingly, sensitivity rates of detection methods used might have been lower here than known from the literature (modified Giemsa staining for *H*. *pylori*, 83.3%; IHC for *H*. *pylori*, 98.8%; IHC for East Asian type of CagA, 93.2%) [15, 34].Especially, immunohistochemistry was not possible in all cases for technical reasons (e.g. too low tissue concentration of the pathogen). Furthermore, the status of *H*. *pylori* was only based on (initial) histology. Other modalities of detection (e.g. rapid urease test) were not routinely performed in all patients that undergo endoscopy at the study institution. Of course, most of these issues could be overcome in a study with prospective patient recruitment. However, given the rather low incidence of gastric cancer in Europe, it would be difficult to recruit a meaningful patient sample within an acceptable time frame at a single institution. Thus, the impressive results of this investigation are an important first step but need further confirmation before drawing general conclusions.

In conclusion, this was the first study to investigate the prevalence of *H*. *pylori* and its respective CagA phenotype in a large retrospective cohort of patients with gastric cancer or duodenal ulcer at a Western tertiary referral institution. The prevalence of *H*. *pylori* decreased dramatically within the past 25 years. The incidence of duodenal ulcer also decreased massively while the incidence of gastric cancer remained stable. Inflammation and atrophy could be observed in both diseases. CagA was highly expressed in *H*. *pylori*. The predominance of the Western subtype raises further questions about its virulence.

## Supporting information

S1 Table Gastric cancer patients (1a) and duodenal ulcer patients (1b).
(DOCX)
