# Supplementary material for: Prevalence of Helicobacter pylori and its CagA subtypes in gastric cancer and duodenal ulcer at an Austrian tertiary referral center over 25 years
Source: PLoS One. 2018 May 29;13(5):e0197695. doi: 10.1371/journal.pone.0197695 (PMC5973618; doi:10.1371/journal.pone.0197695)
Supplement: S1 Table — Gastric cancer patients (1a) and duodenal ulcer patients (1b). (DOCX) [file pone.0197695.s001.docx]

Supplementary table 1a: Gastric cancer patients

| Pat. ID | Sex | Age at endoscopy | Date of gastroscopy | Country of origin | Indication for gastroscopy | Concomittant liver disease | Concomittant antibiotics | CagA Antibody | EA CagA Antibody | Tumor location |
| --- | --- | --- | --- | --- | --- | --- | --- | --- | --- | --- |
| Ga Ca 1 | male | 83 | 10.2010 | Austria | Abdominal pain | - | - | + | - | Antrum |
| Ga Ca 2 | male | 70 | 04.1998 | Yugoslavia | Hematemesis | - | - | + | - | Corpus |
| Ga Ca 3 | male | 67 | 07.2004 | Austria | Hematemesis | - | - | + | - | Antrum |
| Ga Ca 4 | male | 63 | 02.2002 | Austria | Anemia | - | - | + | - | Antrum |
| Ga Ca 5 | male | 62 | 02.1998 | Austria | Cancer screening | - | - | + | - | Antrum |
| Ga Ca 6 | male | 59 | 11.2010 | Yugoslavia | Melena | - | - | + | - | Antrum |
| Ga Ca 7 | female | 55 | 07.1994 | Austria | Cancer screening | - | - | + | - | Corpus |
| Ga Ca 8 | male | 53 | 04.2004 | Turkey | Abdominal pain | - | - | + | - | Corpus |
| Ga Ca 9 | female | 51 | 03.2006 | Turkey | Cancer screening | - | + | + | - | Antrum |
| Ga Ca 10 | female | 47 | 10.2013 | Yugoslavia | Cancer screening | - | + | + | - | Antrum |
| Ga Ca 11 | male | 41 | 03.2013 | China | Loss of appetite | - | - | + | + | Antrum |
| Ga Ca 12 | male | 55 | 04.2006 | Austria | Cancer screening | - | - | - | - | Corpus |
| Ga Ca 13 | male | 36 | 09.1994 | Austria | Melena | - | + | - | - | Antrum |
| Ga Ca 14 | male | 55 | 07.2009 | Austria | Abdominal pain | - | - | + | - | Corpus |

CagA = cytotoxin-associated gene A; EA CagA = East Asian type cytotoxin-associated gene A

Supplementary table 1b: Duodenal ulcer patients

| Pat. ID | Sex | Age at endoscopy | Date of gastroscopy | Country of origin | Indication for gastroscopy | Concomittant liver disease | Concomittant antibiotics | CagA Antibody | EA CagA Antibody |
| --- | --- | --- | --- | --- | --- | --- | --- | --- | --- |
| Du Ulc 1 | male | 84 | 09.1999 | Yugoslavia | Melena | - | + | - | - |
| Du Ulc 2 | male | 53 | 12.2005 | Austria | Cancer screening | - | - | - | - |
| Du Ulc 3 | female | 20 | 06.1998 | Yugoslavia | Cancer screening | - | - | - | - |
| Du Ulc 4 | male | 34 | 07.2006 | Austria | Abdominal pain | - | - | + | - |
| Du Ulc 5 | male | 28 | 01.2002 | Turkey | Abdominal pain | - | - | + | + |
| Du Ulc 6 | male | 73 | 11.1994 | Austria | Abdominal pain | - | - | + | - |
| Du Ulc 7 | male | 65 | 02.2005 | Austria | Abdominal pain | - | + | + | - |
| Du Ulc 8 | male | 57 | 06.2000 | Egypt | Abdominal pain | - | - | + | - |
| Du Ulc 9 | female | 23 | 04.1998 | Kazakhstan | Abdominal pain | - | - | + | - |
| Du Ulc 10 | male | 32 | 10.2000 | China | Abdominal pain | - | - | + | + |
| Du Ulc 11 | male | 41 | 07.2006 | Turkey | Abdominal pain | - | - | + | - |
| Du Ulc 12 | male | 28 | 10.2006 | Yugoslavia | Abdominal pain | - | - | - | - |
| Du Ulc 13 | male | 49 | 12.2005 | Austria | Anemia | + | - | + | - |
| Du Ulc 14 | female | 56 | 07.2006 | Austria | Hematemesis | - | - | + | - |
| Du Ulc 15 | male | 19 | 03.2003 | China | Hematemesis | - | - | + | + |
| Du Ulc 16 | female | 71 | 03.2013 | Austria | Cancer screening | + | + | + | - |
| Du Ulc 17 | male | 62 | 06.2006 | Austria | Cancer screening | - | - | + | - |
| Du Ulc 18 | female | 24 | 12.1995 | Yugoslavia | Loss of appetite | - | - | + | - |

CagA = cytotoxin-associated gene A; EA CagA = East Asian type cytotoxin-associated gene A
